# Global and Regional Voltage and Calcium-Transient Responses to Acute Cardiac Contractility Modulation in Isoproterenol-Treated Rat Hearts with Features Compatible with Early Remodeling

**DOI:** 10.3390/life16091484

**Published:** 2026-09-05

**Authors:** Kaihao Gu, Danyue Mao, Guoliang Hao, Bo Hu, Xiaomei Wu

**Affiliations:** 1 College of Biomedical Engineering, Fudan University, Shanghai 200433, China; kaihaogu@aliyun.com (K.G.); 24210720079@m.fudan.edu.cn (D.M.); 2 Henan Key Laboratory of Cardiac Electrophysiology, Henan Academy of Innovations in Medical Science, Zhengzhou 451100, China; guoliang.hao@epscopelab.com; 3 Henan SCOPE Research Institute of Electrophysiology Co., Ltd., Kaifeng 475000, China; 4 Department of Physiology, Anatomy and Genetics, University of Oxford, Oxford OX1 3PD, UK; 5 Department of Electronic Engineering, School of Information Science and Technology, Fudan University, Shanghai 200438, China; 6 Yiwu Research Institute, Fudan University, Yiwu 322000, China; 7 Shanghai Engineering Research Center of Assistive Devices, Shanghai 200433, China

**Keywords:** cardiac contractility modulation, cardiac electrophysiology, calcium transient, myocardial remodeling, optical mapping, repolarization dispersion, spatial heterogeneity

## Abstract

Background: Cardiac contractility modulation (CCM) improves cardiac performance in heart failure, but its acute spatial effects on myocardial voltage and calcium-transient behavior remain incompletely characterized, particularly in the setting of early myocardial remodeling. Methods: In this exploratory, fixed-sequence study, isolated Langendorff-perfused hearts from Control and isoproterenol (ISO)-treated rats (*n* = 5 per group) underwent simultaneous voltage and calcium optical mapping during a 40-s protocol consisting of pre-stimulation (Base), active CCM stimulation (CCM-ON), and post-stimulation (CCM-OFF) phases. Global metrics were derived across the mapped ventricular surface and regional metrics from predefined regions proximal and distal to the stimulation electrodes. No time-matched sham-stimulation condition was included. Results: Relative optical action-potential (AP) and calcium-transient (CaT) amplitudes were approximately 5–6% higher during CCM-ON and CCM-OFF than during Base in both groups. Global AP duration at 90% repolarization (APD90) showed modest phase-dependent prolongation, whereas global CaT duration at 90% recovery (CTD90) showed modest shortening. Global CTD90 was longer overall in ISO-treated hearts, without a group-by-phase interaction. Three-way repeated-measures analyses identified phase-by-region interactions for CTD90 and AP–CaT delay (the interval from the 50% AP upstroke to the 50% CaT upstroke) and a group-by-region interaction for CTD90, with no significant phase-by-group-by-region interactions. CTD90 spatial dispersion and the absolute proximal–distal difference in AP–CaT delay showed phase effects, whereas mapped-surface APD90 dispersion did not change detectably. Conclusions: During the acute CCM sequence, CaT recovery and AP–CaT timing showed spatially nonuniform phase-associated patterns in Control hearts and ISO-treated hearts with features compatible with early remodeling, without a parallel detectable increase in mapped-surface APD90 dispersion. These exploratory, hypothesis-generating findings support a spatial dissociation between calcium-related temporal responses and mapped-surface APD90 dispersion under the present experimental conditions.

## 1. Introduction

Progressive myocardial remodeling is a central process in the transition from cardiovascular disease to heart failure (HF). It encompasses structural alterations, including cardiomyocyte growth and interstitial fibrosis, as well as changes in cellular electrophysiology and intracellular calcium regulation [1,2,3]. Chronic sympathetic activation and sustained β-adrenergic stimulation can further promote maladaptive electrical, calcium-handling, and contractile remodeling [4]. Importantly, calcium-transient abnormalities may emerge before overt mechanical deterioration. Slowing of cytosolic calcium decline has been demonstrated in mild-to-moderate hypertrophy before detectable slowing of ventricular relaxation [5], while preserved-ejection-fraction disease states can exhibit etiology-dependent abnormalities in diastolic calcium homeostasis and Sarcoplasmic reticulum Ca^2+^ATPase 2a (SERCA2a)-associated signaling [6,7]. Such changes may disturb the temporal coordination between membrane excitation and intracellular calcium activation even when global systolic function remains relatively preserved.

Preventing or delaying pathological remodeling remains an important therapeutic objective. Although contemporary guideline-directed therapies improve clinical outcomes and can promote reverse remodeling, direct and selective targeting of cardiomyocyte calcium-cycling abnormalities remains investigational [8,9]. Conventional positive inotropic therapies can augment contractility but may increase myocardial oxygen demand and arrhythmogenic risk [9]. Cardiac contractility modulation (CCM), a non-excitatory electrical therapy delivered during the absolute refractory period, has emerged as an alternative approach for improving myocardial contractile performance without initiating additional activation [10,11]. Unlike cardiac resynchronization therapy, which primarily modifies ventricular activation and synchrony, CCM is thought to act through downstream cellular and molecular pathways that include modulation of intracellular calcium regulation [10,11].

Because CCM is delivered through localized myocardial electrodes, the simulated electrical field and the resulting biological response may vary spatially across the ventricular myocardium [12]. Clinical studies have demonstrated improvements in functional status and quality of life in selected patients with symptomatic HF [13,14], but the acute global and regional myocardial responses to localized CCM stimulation remain incompletely characterized. This question may be particularly relevant in hearts with early-remodeling features, in which structural and calcium-regulatory abnormalities can coexist with relatively preserved global function [5,6,7]. Moreover, regional electrical and calcium-related heterogeneity may contribute to the development of arrhythmogenic substrates [15,16]. Whether localized calcium-transient responses to CCM are accompanied by a corresponding increase in ventricular repolarization heterogeneity therefore remains unresolved.

To address this issue, we studied hearts from isoproterenol (ISO)-treated rats using a protocol based on previous work demonstrating progressive ventricular remodeling after diffuse catecholamine-induced myocardial injury [17]. Histology and baseline optical mapping were used to determine whether the ISO-treated hearts exhibited features compatible with early remodeling. Using high-resolution dual optical mapping, we simultaneously recorded membrane-voltage and intracellular calcium-transient (CaT) signals across the mapped ventricular surface and within predefined regions relative to the stimulation electrodes. We sought to characterize how voltage behavior, CaT amplitude and recovery, and the temporal relationship between electrical and calcium activation varied across the Base, CCM-ON, and CCM-OFF phases at global and regional levels, and whether spatially heterogeneous calcium-related patterns were accompanied by altered action-potential duration at 90% repolarization (APD90) dispersion. Accordingly, this exploratory mechanistic study aimed to characterize the global and regional organization of phase-associated responses during acute CCM in Control and ISO-treated hearts.

## 2. Materials and Methods

### 2.1. Animals and Experimental Model

This study was designed as an exploratory mechanistic investigation. No formal a priori sample-size calculation was performed. The sample size was selected on the basis of experimental feasibility, and the analyses were intended to characterize response patterns and generate hypotheses rather than provide definitive inference. A total of 10 male Sprague–Dawley rats (weighing 200–240 g, aged 5.5–6.5 weeks) were obtained from SPF Biotechnology Co., Ltd. (Beijing, China). All experimental procedures were reviewed and approved by the Institutional Animal Care and Use Committee of the Henan Academy of Innovations in Medical Science and were conducted in strict accordance with institutional guidelines for animal welfare. The rats were housed in a climate-controlled facility (temperature: 20–26 °C, humidity: 40–70%, pressure differential: 10–20 Pa) under a 12-h light/dark cycle, with ad libitum access to food and water.

Following a brief acclimation period, the rats were randomly assigned to either the Control group (*n* = 5) or the ISO group (*n* = 5) using a simple randomization sequence. To induce catecholamine-associated myocardial injury followed by remodeling, rats in the ISO group received two consecutive subcutaneous injections of ISO (85 mg/kg/day; Sigma-Aldrich, Burlington, MA, USA) with a 24-h interval [17]. Both groups were subsequently maintained under standard conditions for 4 weeks prior to the ex vivo experiments. No animal mortality was observed throughout the induction and maintenance periods.

### 2.2. Heart Isolation and Langendorff Perfusion

Rats were intraperitoneally injected with heparin sodium (3125 U/kg; BIOISCO Biotechnology, Lianyungang, China) to prevent blood coagulation. Fifteen minutes later, the animals were anesthetized with isoflurane (RWD Life Science, Shenzhen, China) and euthanized by cervical dislocation to ensure rapid and humane sacrifice. The hearts were rapidly excised and immediately immersed in oxygenated (95% O_2_/5% CO_2_) Krebs–Henseleit (KH) solution (comprising 119 mM NaCl, 4 mM KCl, 1.8 mM CaCl_2_, 1 mM MgCl_2_, 1.2 mM NaH_2_PO_4_, 25 mM NaHCO_3_, and 10 mM Glucose; all purchased from Sigma-Aldrich).

The aorta was cannulated and mounted onto a Langendorff apparatus. Retrograde perfusion with the KH solution was maintained at a constant flow rate of 8 mL/min and a physiological temperature of 37 ± 0.5 °C. After stabilization, hearts were evaluated according to predefined inclusion criteria including: (1) maintenance of stable perfusion conditions under constant-flow mode; (2) absence of persistent, irreversible ventricular tachyarrhythmias; and (3) a high signal-to-noise ratio for subsequent high-fidelity optical recordings. No hearts met exclusion criteria. To eliminate motion artifacts during optical mapping, the electromechanical uncoupler Blebbistatin (Abcam, Cambridge, UK) was continuously perfused. The hearts were then sequentially loaded with Pluronic F-127 (Sigma-Aldrich) and the intracellular calcium indicator Rhod-2 AM (Abcam) for 10 and 15 min, respectively. Finally, the voltage-sensitive dye RH237 (Abcam) was added to the perfusate to complete the dual-mapping preparation.

### 2.3. Pacing and CCM Stimulation Protocols

Each experiment consisted of a continuous 40-s recording session to systematically capture the kinetic transitions of myocardial responses. Each session followed a fixed predefined temporal protocol: an initial 5-s baseline phase (Base, standard pacing only), followed by a 20-s active stimulation phase (CCM-ON), and concluding with a 15-s stimulus withdrawal phase (CCM-OFF). A separate time-matched pacing-only sham-stimulation session was not performed.

Electrical stimulation was delivered using a programmable stimulator (VCS-3002, MappingLab, Oxford, UK) capable of both standard pacing and specialized CCM stimuli. An S1–S2 pacing protocol was initially applied to determine the effective refractory period of the isolated rat hearts. The baseline pacing frequency was set at 5 Hz. To simulate the clinical application of CCM, two needle electrodes were superficially inserted into the epicardium of the interventricular septum with an inter-electrode distance of 1 cm. The CCM stimulus was triggered approximately 25 ms after local detection, based on the measured refractory period. No additional propagated activation was observed, supporting exclusive delivery within the absolute refractory period. The CCM signal in each cardiac cycle consisted of two monophasic pulses, each with a pulse amplitude of 7.5 V, a phase duration of 5 ms and an interphase interval of 5 ms.

### 2.4. Optical Mapping Data Acquisition

Optical mapping of the isolated hearts was performed using a dual-LED light source centered at 530 ± 25 nm with a band-pass filter at 511–551 nm (LEDC-2001, MappingLab). Emitted fluorescent photons were optically filtered and separated to independently capture transmembrane voltage signals and intracellular calcium transient signals. The signals were acquired simultaneously using two high-speed CMOS cameras (OMS-PCIE-2002, MappingLab) operating at a sampling rate of 900 Hz. All optical signals were processed using OMapScope5.9.45 software (MappingLab).

### 2.5. Signal Processing and Mapping Metrics

To standardize signal sampling across experimental phases and minimize transient onset effects, the final three consecutive paced beats from each phase—pre-stimulation (Base), active CCM stimulation (CCM-ON), and post-stimulation (CCM-OFF)—were selected for analysis. Voltage- and calcium-sensitive fluorescence signals were analyzed as fractional fluorescence changes (ΔF/F).

Quantitative analyses were performed at mapped-surface and predefined regional levels. For pixel-based measurements, global values were calculated by averaging valid pixel-wise values across the mapped ventricular surface within each heart and experimental phase. Global metrics included APD90 (ms), calcium-transient duration at 90% recovery (CTD90, ms), action potential (AP) and CaT upstroke times (ms), total ventricular activation time (ms) and total CaT activation time (ms). Total ventricular activation time was defined as the interval between the earliest and latest AP activation times across the mapped surface, whereas total CaT activation time was defined as the corresponding interval between the earliest and latest CaT activation times.

Global AP and CaT amplitudes were first measured from the ΔF/F waveforms. For each heart, the mean amplitudes during CCM-ON and CCM-OFF were then divided by the corresponding mean amplitude during the Base phase. Base was therefore assigned a value of 1.0, and CCM-ON and CCM-OFF values were expressed as relative amplitude ratios. This phase-level normalization was performed separately for the AP and CaT signals within each heart.

Regional analyses focused on CTD90 and AP–CaT delay within six predefined circular regions of interest (ROIs) of identical size. ROI locations were determined before quantitative extraction according to their geometric relationship to the stimulation electrodes and were applied consistently across hearts. Because the epicardial CCM electrodes partially obscured the optical field and could cause local tissue injury at the insertion sites, all ROIs were positioned within unobstructed mapped myocardium. Three ROIs were assigned to the proximal region: two ROI centers were located approximately 2 mm from the respective stimulation electrodes, and the third was positioned midway between them. Three corresponding distal ROIs were placed parallel to the proximal ROIs, with an approximate proximal-to-distal inter-center distance of 5.5 mm, representing the greatest feasible separation while retaining all ROIs within the mapped myocardial surface. Measurements from the three ROIs within each region were averaged within each heart and phase to obtain representative proximal and distal values.

The temporal relationship between electrical and calcium activation was quantified as AP–CaT delay, defined as the interval between the 50% amplitude point of the AP upstroke and the corresponding 50% amplitude point of the CaT upstroke. To assess spatial heterogeneity, the spatial standard deviations (SDs) of pixel-wise APD90 and CTD90 were calculated across the valid mapped ventricular surface for each heart and phase. A complementary regional heterogeneity metric was calculated as the absolute difference between the mean proximal and distal AP–CaT delays.

### 2.6. Statistical Analysis

Statistical analyses were performed using GraphPad Prism version 10.6.1 (GraphPad Software, Boston, MA, USA). Each heart was treated as the experimental unit and biological replicate (*n* = 5 per group). Data are presented as mean ± standard error of the mean (SEM), with individual-heart values shown. All *t* tests and pairwise comparisons were two-sided, and *p* < 0.05 was considered statistically significant. For multiplicity adjustment, a multiple-comparison family was the prespecified set of related comparisons addressing one statistical question for a given metric; adjustments were applied within each family, not across metrics.

Baseline between-group comparisons used to characterize the ISO-treated phenotype were performed using two-tailed unpaired Welch’s *t* tests. Between-group effects are reported as the mean difference (ISO − Control) with its 95% confidence interval (CI) and as Hedges’ *g* with its 95% CI. Hedges’ *g* was calculated using the pooled standard deviation and the exact small-sample J correction.

For amplitude analyses, the CCM-ON and CCM-OFF amplitude ratios were compared with the hypothetical Base reference value of 1.0 within each group using two-tailed one-sample *t* tests. For each amplitude metric and group, the two reference-value comparisons (CCM-ON vs. 1.0 and CCM-OFF vs. 1.0) were treated as one family and adjusted using the Šídák method. Between-group differences in amplitude ratios were assessed using two-tailed unpaired Welch’s *t* tests. For each amplitude metric, the two between-group comparisons (Control vs. ISO at CCM-ON and Control vs. ISO at CCM-OFF) were treated as one family and adjusted using the Šídák method. Between-group effects are reported as Hedges’ *g* with 95% CIs.

Global duration, activation-time, and spatial-heterogeneity metrics were analyzed using two-way repeated-measures ANOVA, with group (Control vs. ISO) as the between-subject factor and phase (Base, CCM-ON, and CCM-OFF) as the repeated within-subject factor. Regional metrics were analyzed using three-way repeated-measures ANOVA, with group as the between-subject factor and phase and region (proximal vs. distal) as repeated within-subject factors. The regional models therefore evaluated all three main effects, the three two-way interactions, and the group-by-phase-by-region interaction. The Geisser–Greenhouse correction was applied to effects involving phase. ANOVA results are reported as $F(df_{num},\;{df}_{den})$, where $df_{num}$ and ${df}_{den}$ denote the numerator and denominator degrees of freedom, respectively, with exact *p* values and partial eta squared (partial η^2^) for each effect. Geisser–Greenhouse-corrected degrees of freedom and *p* values are reported for effects involving phase when applicable.

Prespecified within-group phase comparisons comprised Base vs. CCM-ON, Base vs. CCM-OFF, and CCM-ON vs. CCM-OFF. For each metric and group, these three comparisons were treated as one family and adjusted using the Tukey method. Prespecified phase-specific between-group comparisons comprised Control vs. ISO at Base, Control vs. ISO at CCM-ON, and Control vs. ISO at CCM-OFF. For each metric, these three comparisons were treated as one family and adjusted using the Šídák method. Region-specific follow-up comparisons were used to decompose the regional models. Within each proximal or distal region, the same comparison families were applied: the three phase comparisons within each group were Tukey-adjusted, and the three Control vs. ISO comparisons across phases were Šídák-adjusted. To express regional effect direction in the original unit, directed proximal-minus-distal contrasts and their *t*-based 95% CIs were also calculated; these CIs were used for estimation and were not treated as an additional multiplicity-adjusted post hoc family. Adjusted *p* values and 95% CIs are reported for prespecified comparisons. There were no missing values or post hoc exclusions.

The isolated-heart preparation and CCM delivery were performed by non-author personnel at an external research facility who were aware of group allocation. Raw optical-mapping data were subsequently provided to the authors under coded identifiers, and signal processing, ROI selection, metric extraction, and all statistical analyses were completed using coded group labels without knowledge of which label represented the Control or ISO group; the allocation code was disclosed only after all analyses had been finalized. Histological specimens were processed and stained with Masson’s trichrome at a separate external facility by personnel unaware of group allocation. The stained sections were returned without interpretation and were subsequently linked to their experimental groups by the authors before qualitative histological assessment; therefore, the histological interpretation itself was not blinded.

## 3. Results

### 3.1. Baseline Structural and Electrophysiological Characteristics of ISO-Treated Hearts

**Figure 1:**
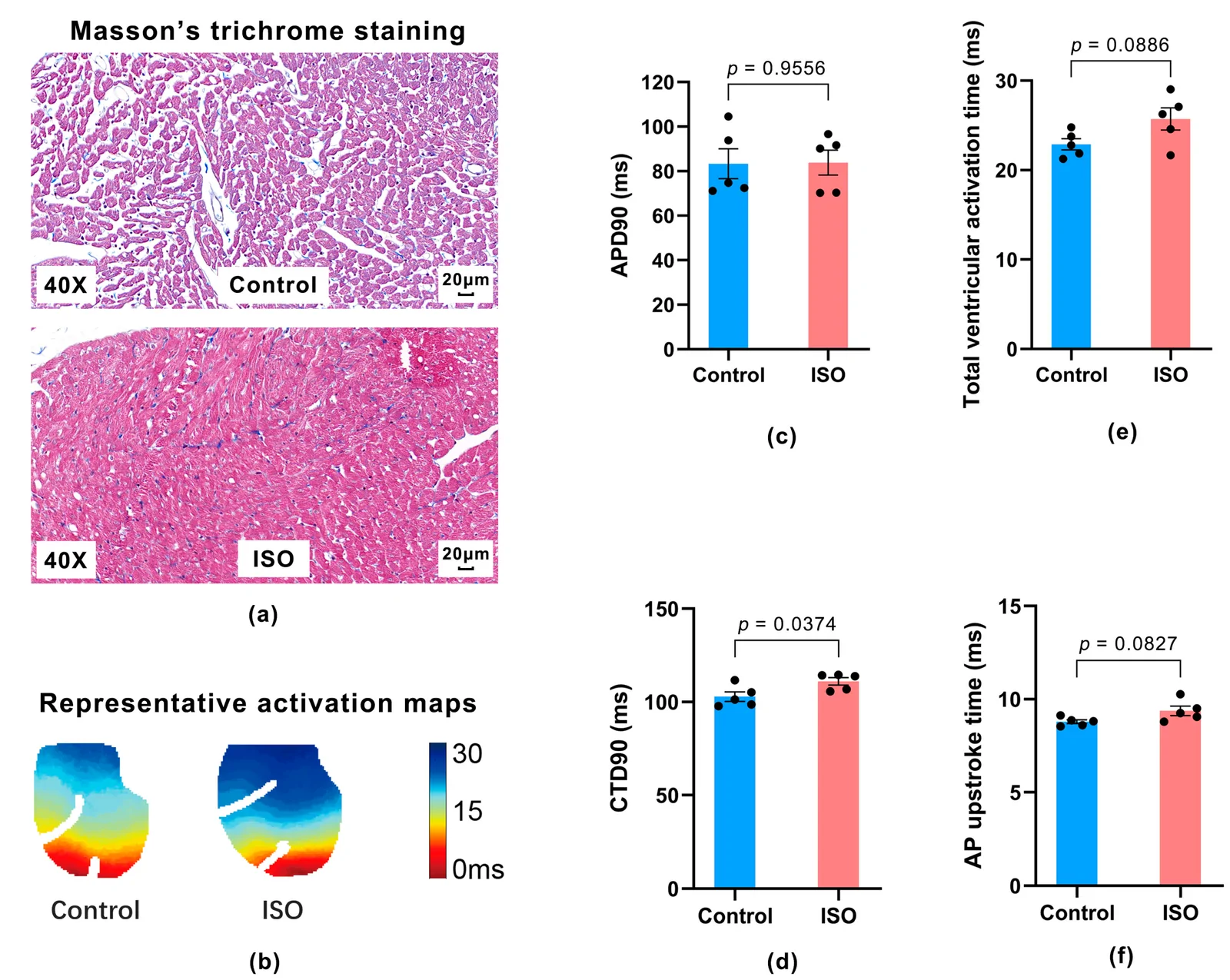
Representative ventricular histology and baseline electrophysiology. (**a**) Representative Masson’s trichrome-stained ventricular sections from Control and isoproterenol (ISO)-treated hearts. The ISO-treated example shows mild interstitial collagen staining and qualitative alterations in myocardial architecture. (**b**) Representative activation maps across the mapped ventricular surface. The ISO-treated example shows later completion of ventricular activation. (**c**,**d**) Baseline global action potential duration at 90% repolarization (APD90; (**c**)) and calcium-transient duration at 90% recovery (CTD90 (**d**)). (**e**,**f**) Total ventricular activation time (**e**) and action potential (AP) upstroke time (**f**). Data are presented as mean ± standard error of the mean (SEM), with individual hearts shown (*n* = 5 per group). Between-group comparisons were performed using two-tailed unpaired Welch’s *t* tests.

Representative Masson’s trichrome-stained sections showed mild interstitial collagen staining and qualitative alterations in myocardial architecture in ISO-treated hearts compared with Control hearts (Figure 1a). Representative activation maps suggested later completion of activation across the mapped ventricular surface in ISO-treated hearts (Figure 1b). At baseline, global APD90 did not differ significantly between Control and ISO-treated hearts (83.32 ± 6.71 versus 83.83 ± 5.62 ms; mean difference = 0.50 ms; 95% CI for the mean difference: −19.80 to 20.81 ms; *p* = 0.9556; Hedges’ *g* = 0.033, 95% CI for *g*: −1.087 to 1.151; Figure 1c). In contrast, global CTD90 was longer in ISO-treated hearts (102.91 ± 2.54 versus 111.04 ± 1.98 ms; mean difference = 8.13 ms; 95% CI for the mean difference: 0.62 to 15.64 ms; *p* = 0.0374; Hedges’ *g* = 1.440, 95% CI for *g*: 0.092 to 2.725; Figure 1d), corresponding to an increase of approximately 7.9%. Total ventricular activation time was 22.89 ± 0.64 ms in Control and 25.72 ± 1.24 ms in ISO-treated hearts (mean difference = 2.83 ms; 95% CI for mean difference = −0.58 to 6.25 ms; *p* = 0.0886; Hedges’ *g* = 1.160, 95% CI for *g*: −0.119 to 2.383), and AP upstroke time was 8.79 ± 0.10 versus 9.38 ± 0.25 ms (mean difference = 0.58 ms; 95% CI for mean difference = −0.11 to 1.27 ms; *p* = 0.0827; Hedges’ *g* = 1.218, 95% CI for *g*: −0.075 to 2.452; Figure 1e,f).

Thus, the ISO-treated hearts exhibited qualitative structural alterations and prolonged baseline CTD90, whereas baseline APD90 did not differ detectably. In addition, total ventricular activation time and AP upstroke time were numerically longer in ISO-treated hearts. These findings supported characterization of the ISO-treated hearts as an experimental phenotype with features compatible with early myocardial remodeling and provided the context for assessing acute CCM responses.

### 3.2. Global Voltage and Calcium-Transient Responses to Acute CCM

**Figure 2:**
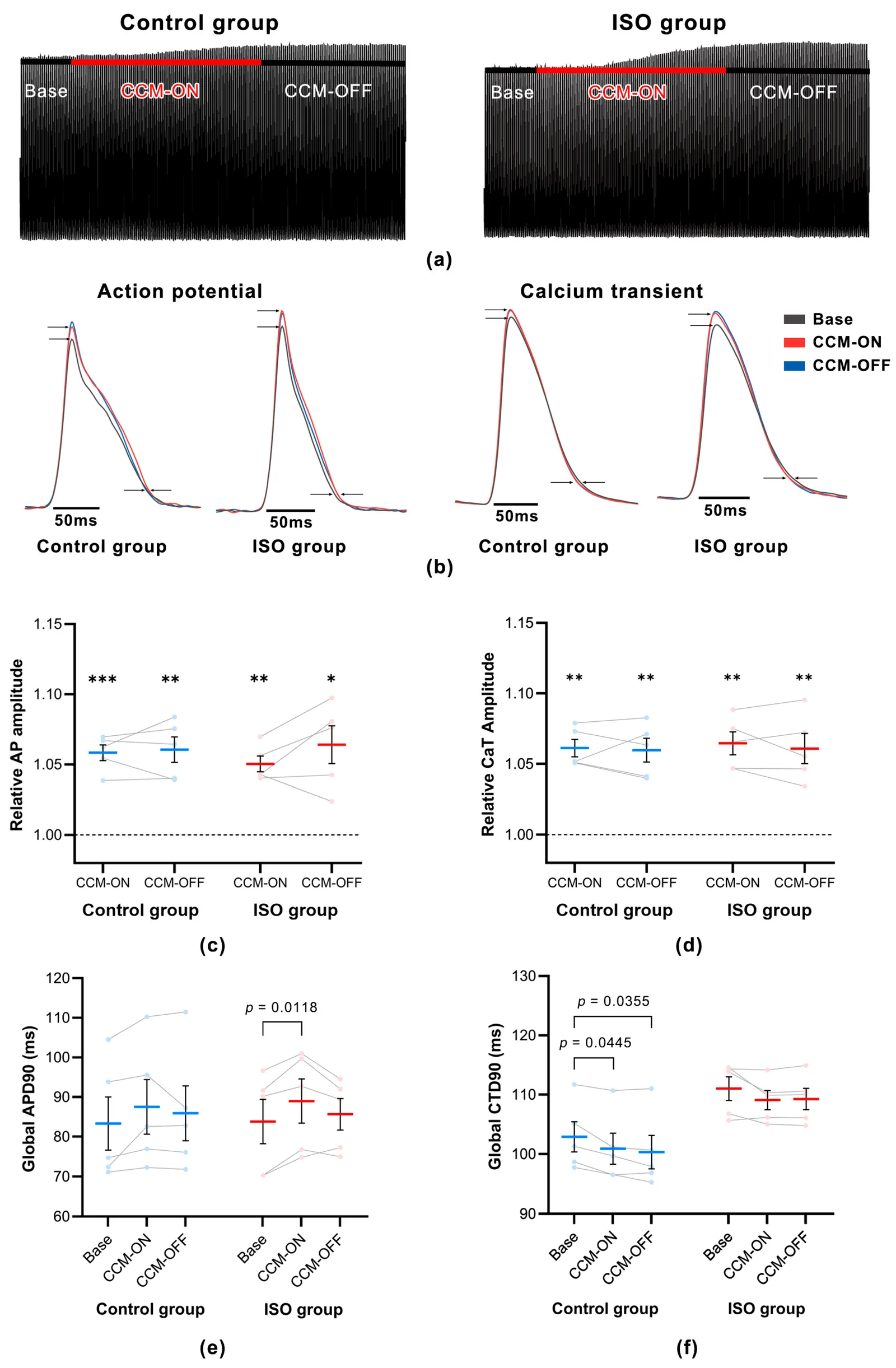
Global optical responses during acute cardiac contractility modulation. (**a**) Representative continuous calcium-transient (CaT) fluorescence recordings from Control and ISO-treated hearts during Base, CCM-ON, and CCM-OFF. (**b**) Representative superimposed optical action-potential (AP) and CaT waveforms. (**c**,**d**) Relative optical AP amplitude (**c**) and CaT amplitude (**d**), normalized to the corresponding mean Base value within each heart. For each metric and group, one-sample comparisons with 1.0 were Šídák-adjusted across CCM-ON and CCM-OFF. For each metric, phase-specific between-group comparisons using Welch’s *t* tests were Šídák-adjusted across CCM-ON and CCM-OFF. (**e**,**f**) Global APD90 (**e**) and CTD90 (**f**). APD90 and CTD90 were analyzed using two-way repeated-measures ANOVA. For each metric, within-group phase comparisons were Tukey-adjusted within each group, and phase-specific between-group comparisons were Šídák-adjusted across the three phases. Data are presented as mean ± SEM, with individual-heart values connected across phases (*n* = 5 per group). Only statistically significant comparisons are annotated. For panels c and d, * *p* < 0.05, ** *p* < 0.01, and *** *p* < 0.001 versus 1.0 after adjustment. Exact ANOVA and pairwise-comparison results are reported in Section 3.2.

To characterize global responses across the fixed acute CCM sequence, optical signals aggregated across the mapped ventricular surface were analyzed (Figure 2). Representative continuous recordings displayed progressively higher CaT fluorescence amplitude across the sequence in both groups, with the higher amplitude remaining evident during CCM-OFF relative to Base (Figure 2a). Representative superimposed waveforms showed higher CaT amplitude and earlier recovery during CCM-ON and CCM-OFF than during Base, whereas AP waveforms showed higher optical amplitude and a broader plateau with comparatively smaller changes in terminal repolarization (Figure 2b). During CCM-OFF, representative AP morphology shifted toward Base, whereas CaT waveforms remained broadly similar to those during CCM-ON.

Quantitative analysis showed that optical AP ΔF/F amplitude ratios remained above the Base reference during CCM-ON and CCM-OFF after Šídák adjustment. In Control hearts, the values were 5.84% above Base during CCM-ON (95% CI: 4.29% to 7.39%; adjusted *p* = 0.0009) and 6.05% above Base during CCM-OFF (95% CI: 3.53% to 8.58%; adjusted *p* = 0.0053). In ISO-treated hearts, the corresponding values were 5.04% (95% CI: 3.49% to 6.59%; adjusted *p* = 0.0017) and 6.41% (95% CI: 2.68% to 10.14%; adjusted *p* = 0.0176) above Base, respectively (Figure 2c). At CCM-ON, the ISO-minus-Control difference was −0.00798 ratio units (95% CI: −0.02617 to 0.01021; adjusted *p* = 0.5663; Hedges’ *g* = −0.577, 95% CI for *g*: −1.714 to 0.592). At CCM-OFF, the corresponding difference was 0.00358 ratio units (95% CI: −0.03477 to 0.04193; adjusted *p* = 0.9717; Hedges’ *g* = 0.126, 95% CI for *g*: −0.999 to 1.243).

CaT ΔF/F amplitude showed a similar pattern. In Control hearts, the values were 6.12% above Base during CCM-ON (95% CI: 4.41% to 7.84%; adjusted *p* = 0.0012) and 5.97% above Base during CCM-OFF (95% CI: 3.64% to 8.31%; adjusted *p* = 0.0042). In ISO-treated hearts, the corresponding values were 6.46% (95% CI: 4.21% to 8.71%; adjusted *p* = 0.0027) and 6.09% (95% CI: 3.12% to 9.06%; adjusted *p* = 0.0094) above Base, respectively (Figure 2d). At CCM-ON, the ISO-minus-Control mean difference was 0.00340 ratio units (95% CI: −0.02038 to 0.02718; adjusted *p* = 0.9364; Hedges’ *g* = 0.191, 95% CI for *g*: −0.938 to 1.307). At CCM-OFF, the corresponding difference was 0.00114 ratio units (95% CI: −0.03054 to 0.03282; adjusted *p* = 0.9958; Hedges’ *g* = 0.048, 95% CI for *g*: −1.073 to 1.166).

For global APD90, two-way ANOVA showed a phase effect (*F*(1.421, 11.37) = 6.234, *p* = 0.0212, partial η^2^ = 0.438), but neither the group effect (*F*(1, 8) = 0.0047, *p* = 0.9470, partial η^2^ = 0.001) nor the group-by-phase interaction (*F*(1.421, 11.37) = 0.211, *p* = 0.7384, partial η^2^ = 0.026) was significant (Figure 2e). In Control hearts, APD90 increased from 83.32 ± 6.71 ms at Base to 87.52 ± 6.90 ms during CCM-ON (mean change = 4.20 ms; 95% CI: −1.83 to 10.23 ms; adjusted *p* = 0.1388). In ISO-treated hearts, APD90 increased from 83.83 ± 5.62 to 89.00 ± 5.57 ms (mean change = 5.17 ms; 95% CI: 1.81 to 8.53 ms; adjusted *p* = 0.0118). During CCM-OFF, values moved toward Base in both groups (Control, 85.90 ± 6.93 ms; ISO-treated, 85.65 ± 3.99 ms), and neither OFF-versus-Base comparison was significant (adjusted *p* = 0.6754 and 0.5784).

Global CTD90 showed group (*F*(1, 8) = 7.107, *p* = 0.0285, partial η^2^ = 0.470) and phase effects (*F*(1.226, 9.807) = 11.95, *p* = 0.0048, partial η^2^ = 0.599), without a group-by-phase interaction (*F*(1.226, 9.807) = 0.418, *p* = 0.5735, partial η^2^ = 0.050; Figure 2f). In Control hearts, CTD90 decreased from 102.91 ± 2.54 ms at Base to 100.91 ± 2.60 ms during CCM-ON (mean change = −2.01 ms; 95% CI: −3.94 to −0.07 ms; adjusted *p* = 0.0445) and to 100.35 ± 2.81 ms during CCM-OFF (mean change = −2.57 ms; 95% CI: −4.87 to −0.26 ms; adjusted *p* = 0.0355). In ISO-treated hearts, CTD90 decreased from 111.04 ± 1.98 ms to 109.11 ± 1.63 and 109.28 ± 1.79 ms, but the corresponding comparisons were not significant (adjusted *p* = 0.2311 and 0.2963). Although the group main effect indicated a longer overall CTD90 in ISO-treated hearts, the Šídák-adjusted ISO-minus-Control comparisons at Base, CCM-ON, and CCM-OFF did not individually reach 0.05 (mean differences = 8.13, 8.20, and 8.94 ms; 95% CIs: −1.70 to 17.96, −1.48 to 17.88, and −1.53 to 19.40 ms; adjusted *p* = 0.1080, 0.0962, and 0.0937, respectively).

Because CCM was delivered locally, phase- and group-dependent regional patterns of CTD90 and AP–CaT delay were next examined in a three-factor model.

### 3.3. Regional Patterns of CTD90 and AP–CaT Delay During Acute CCM

**Figure 3:**
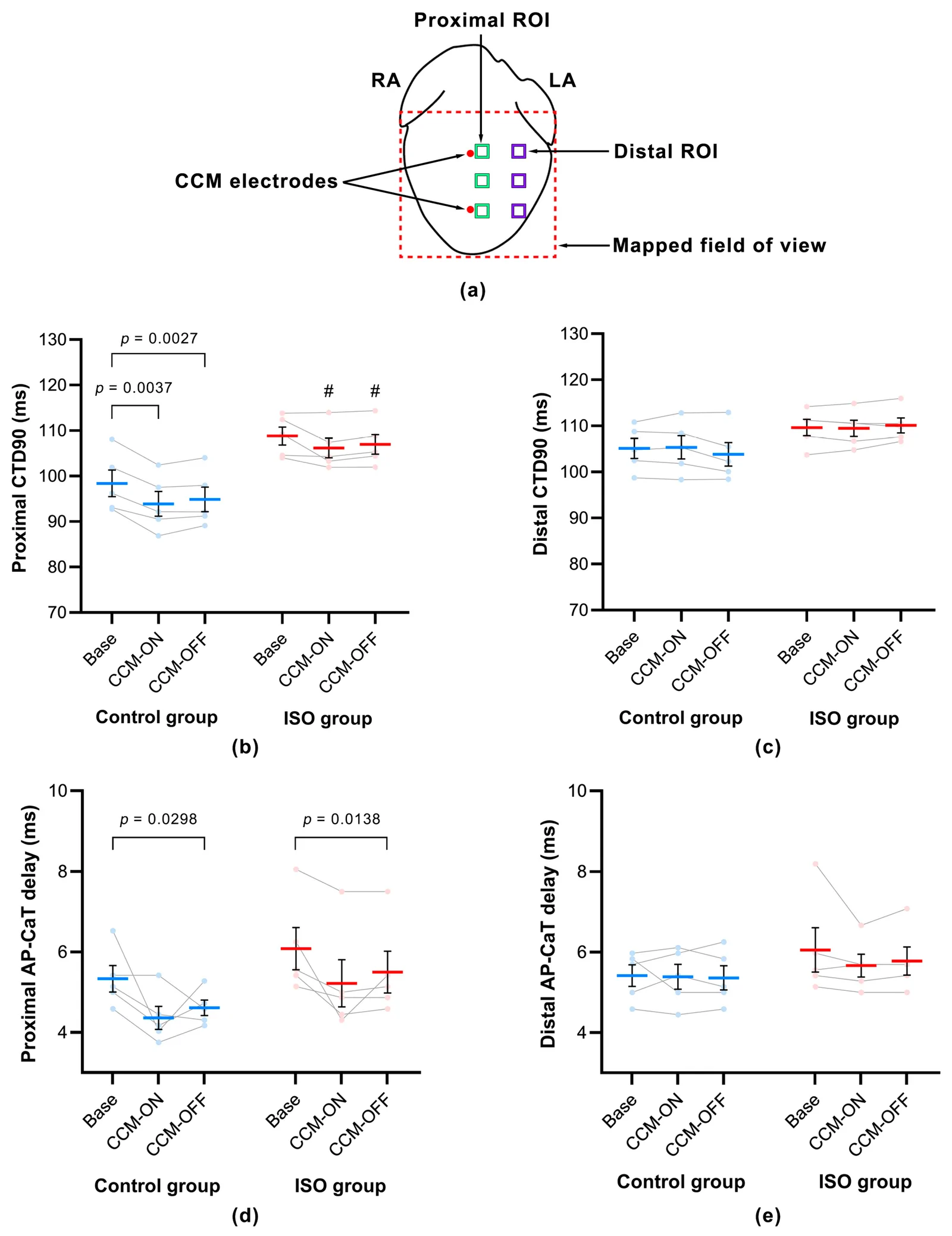
Regional CTD90 and AP–CaT delay. (**a**) Schematic of CCM electrode locations and predefined proximal and distal regions of interest (ROIs). Red circles indicate stimulation electrodes, green squares proximal ROIs, purple squares distal ROIs, and the dashed red outline the mapped field of view. RA, right atrium; LA, left atrium. (**b**,**c**) CTD90 in proximal (**b**) and distal (**c**) ROIs. (**d**,**e**) AP–CaT delay in proximal (**d**) and distal (**e**) ROIs. Regional data were analyzed using three-way repeated-measures ANOVA with group, phase, and region, followed by prespecified region-specific comparisons. Within-group phase comparisons were Tukey-adjusted within each group, and between-group comparisons were Šídák-adjusted across phases. Data are mean ± SEM with individual hearts connected across phases (*n* = 5 per group). Brackets denote significant within-group phase comparisons; # *p* < 0.05 for ISO versus Control at the corresponding phase after adjustment. Exact results are reported in Section 3.3.

Three-way repeated-measures ANOVA of CTD90 showed main effects of phase (*F*(1.352, 10.81) = 10.10, *p* = 0.0060, partial η^2^ = 0.558), group (*F*(1, 8) = 7.074, *p* = 0.0288, partial η^2^ = 0.469), and region (*F*(1, 8) = 69.58, *p* < 0.0001, partial η^2^ = 0.897). The phase-by-region interaction (*F*(1.217, 9.734) = 22.30, *p* = 0.0006, partial η^2^ = 0.736) and group-by-region interaction (*F*(1, 8) = 23.11, *p* = 0.0013, partial η^2^ = 0.743) were also significant, demonstrating that phase changes and group differences depended on region. The phase-by-group interaction was not significant (*F*(1.352, 10.81) = 2.032, *p* = 0.1827, partial η^2^ = 0.203), and there was no phase-by-group-by-region interaction (*F*(1.217, 9.734) = 2.836, *p* = 0.1203, partial η^2^ = 0.262; Figure 3b,c).

In Control hearts, proximal CTD90 decreased from 98.40 ± 2.93 ms at Base to 93.90 ± 2.74 ms during CCM-ON (mean change = −4.50 ms; 95% CI: −6.64 to −2.36 ms; adjusted *p* = 0.0037) and remained shorter during CCM-OFF (94.88 ± 2.71 ms; mean change = −3.52 ms; 95% CI: −5.04 to −1.99 ms; adjusted *p* = 0.0027). Proximal CTD90 in ISO-treated hearts changed from 108.81 ± 1.99 to 106.16 ± 2.15 and 106.98 ± 2.15 ms; neither comparison with Base was significant. The ISO-minus-Control proximal difference was 10.41 ms at Base (95% CI: −0.62 to 21.44 ms; adjusted *p* = 0.0638), 12.26 ms during CCM-ON (95% CI: 1.66 to 22.86 ms; adjusted *p* = 0.0253), and 12.10 ms during CCM-OFF (95% CI: 1.57 to 22.63 ms; adjusted *p* = 0.0261). Distal CTD90 varied less across phases, and no phase-specific between-group comparison was significant. Directed proximal-minus-distal contrasts were negative in Control hearts at Base, CCM-ON, and CCM-OFF (−6.71 ms, 95% CI: −10.08 to −3.34; −11.45 ms, 95% CI: −14.81 to −8.08; and −8.96 ms, 95% CI: −10.99 to −6.92, respectively). The corresponding ISO estimates were −0.83 ms (95% CI: −3.40 to 1.74), −3.33 ms (95% CI: −6.82 to 0.15), and −3.12 ms (95% CI: −5.84 to −0.41). Relative to Base, the Control regional contrast shifted by −4.73 ms during CCM-ON (95% CI: −7.21 to −2.25), supporting a greater phase-related shortening proximally than distally.

For AP–CaT delay, three-way ANOVA showed a phase effect (*F*(1.528, 12.22) = 13.74, *p* = 0.0013, partial η^2^ = 0.632), a region effect (*F*(1, 8) = 7.955, *p* = 0.0225, partial η^2^ = 0.499), and a phase-by-region interaction (*F*(1.934, 15.47) = 3.735, *p* = 0.0487, partial η^2^ = 0.318). The group effect (*F*(1, 8) = 1.632, *p* = 0.2372, partial η^2^ = 0.169), phase-by-group interaction (*p* = 0.7937, partial η^2^ = 0.020), group-by-region interaction (*p* = 0.2339, partial η^2^ = 0.172), and three-way interaction (*F*(1.934, 15.47) = 0.434, *p* = 0.6493, partial η^2^ = 0.051) were not significant (Figure 3d,e).

Proximal AP–CaT delay in Control hearts decreased from 5.33 ± 0.33 ms at Base to 4.36 ± 0.29 ms during CCM-ON and 4.61 ± 0.19 ms during CCM-OFF; the OFF-versus-Base difference was −0.72 ms (95% CI: −1.34 to −0.11 ms; adjusted *p* = 0.0298), whereas the ON-versus-Base comparison was not significant. In ISO-treated hearts, proximal delay decreased from 6.08 ± 0.53 to 5.22 ± 0.58 and 5.50 ± 0.52 ms; the OFF-versus-Base difference was −0.58 ms (95% CI: −0.98 to −0.19 ms; adjusted *p* = 0.0138). Distal values changed less (Control: 5.42 ± 0.27, 5.39 ± 0.31, and 5.36 ± 0.30 ms; ISO-treated: 6.06 ± 0.55, 5.67 ± 0.28, and 5.78 ± 0.35 ms), and no distal phase comparison was significant. The directed proximal-minus-distal contrast was more negative during CCM-ON and CCM-OFF than at Base in both groups. Control estimates were −0.08 ms at Base (95% CI: −0.85 to 0.68), −1.03 ms during CCM-ON (95% CI: −2.19 to 0.13), and −0.75 ms during CCM-OFF (95% CI: −1.37 to −0.13); ISO estimates were 0.03 ms (95% CI: −0.60 to 0.65), −0.44 ms (95% CI: −1.47 to 0.58), and −0.28 ms (95% CI: −1.00 to 0.44), respectively. Global AP–CaT delay showed no phase effect (Table A1).

These interaction results establish spatially nonuniform CTD90 and AP–CaT timing responses without evidence that the phase-by-region pattern differed between Control and ISO-treated hearts. We next evaluated whether these regional patterns were accompanied by broader changes in mapped-surface heterogeneity.

### 3.4. Spatial Variation in CTD90, AP–CaT Delay, and APD90 During Acute CCM

**Figure 4:**
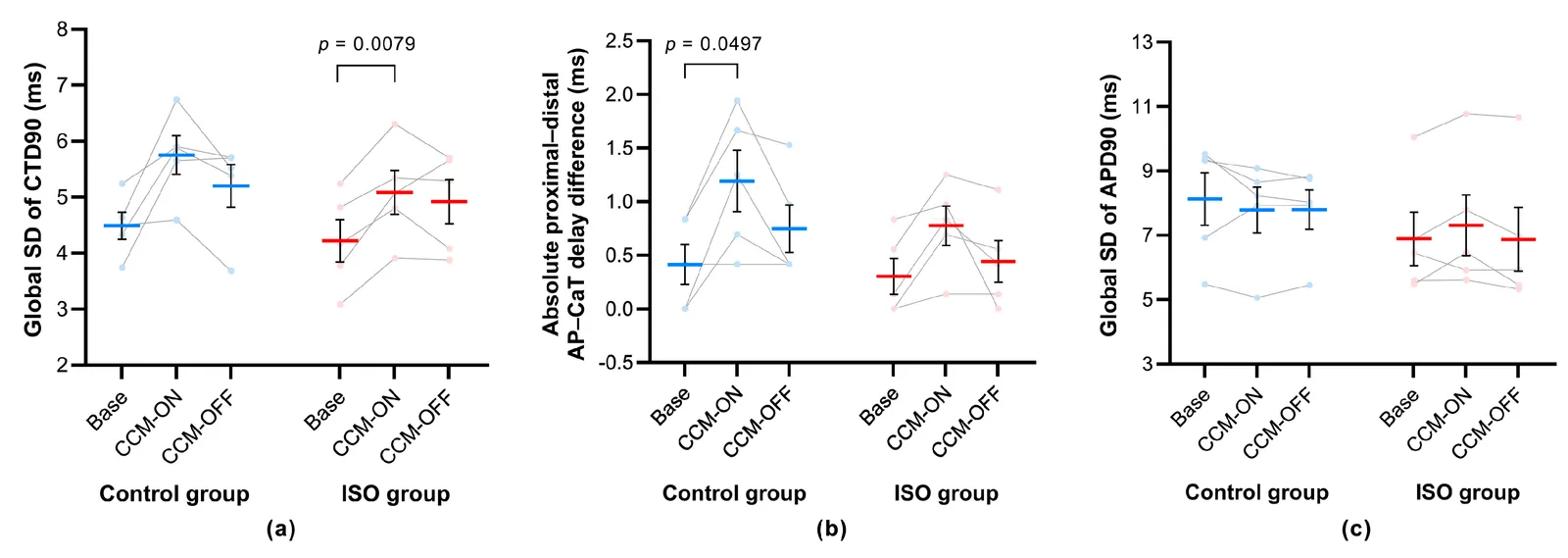
Spatial heterogeneity of CTD90, AP–CaT delay, and APD90. (**a**) Spatial standard deviation (SD) of pixel-wise CTD90 across the mapped ventricular surface. (**b**) Absolute difference in AP–CaT delay between proximal and distal ROIs. (**c**) Spatial SD of pixel-wise APD90. Data were analyzed using two-way repeated-measures ANOVA. Within-group phase comparisons were Tukey-adjusted within each group, and between-group comparisons were Šídák-adjusted across phases. Data are mean ± SEM with individual hearts connected across phases (*n* = 5 per group). Only statistically significant within-group comparisons are annotated. Exact results are reported in Section 3.4.

To further characterize spatial variation during acute CCM, we evaluated the mapped-surface spatial SDs of CTD90 and APD90 and the absolute proximal–distal difference in AP–CaT delay (Figure 4). The SD metrics quantify pixel-wise dispersion across the mapped ventricular surface, whereas the absolute AP–CaT delay difference quantifies the magnitude, but not the direction, of regional separation between the two predefined ROI sets.

CTD90 spatial SD showed a phase effect (*F*(1.449, 11.59) = 11.96, *p* = 0.0027, partial η^2^ = 0.599), but neither the group effect (*F*(1, 8) = 0.850, *p* = 0.3837, partial η^2^ = 0.096) nor the group-by-phase interaction (*F*(1.449, 11.59) = 0.523, *p* = 0.5497, partial η^2^ = 0.061) was significant (Figure 4a). In Control hearts, CTD90 spatial SD increased from 4.49 ± 0.24 ms at Base to 5.75 ± 0.34 ms during CCM-ON (mean change = 1.26 ms; 95% CI: −0.10 to 2.63 ms; adjusted *p* = 0.0637) and was 5.20 ± 0.38 ms during CCM-OFF. In ISO-treated hearts, it increased from 4.22 ± 0.38 to 5.09 ± 0.39 ms during CCM-ON (mean change = 0.87 ms; 95% CI: 0.36 to 1.37 ms; adjusted *p* = 0.0079) and remained numerically elevated during CCM-OFF (4.92 ± 0.39 ms; adjusted *p* = 0.1950 versus Base).

The absolute proximal–distal AP–CaT delay difference also showed a phase effect (*F*(1.968, 15.75) = 11.18, *p* = 0.0010, partial η^2^ = 0.583), without a group effect (*F*(1, 8) = 1.192, *p* = 0.3067, partial η^2^ = 0.130) or group-by-phase interaction (*F*(1.968, 15.75) = 0.672, *p* = 0.5227, partial η^2^ = 0.077; Figure 4b). In Control hearts, the absolute difference increased from 0.42 ± 0.19 ms at Base to 1.19 ± 0.29 ms during CCM-ON (mean change = 0.78 ms; 95% CI: 0.001 to 1.55 ms; adjusted *p* = 0.0497) and then decreased to 0.75 ± 0.22 ms during CCM-OFF. In ISO-treated hearts, the values were 0.31 ± 0.17, 0.78 ± 0.18, and 0.44 ± 0.19 ms; the ON-versus-Base change was 0.47 ms (95% CI: −0.013 to 0.96 ms; adjusted *p* = 0.0543). No between-group comparison was significant.

APD90 spatial SD showed no phase effect (*F*(1.389, 11.11) = 0.639, *p* = 0.4913, partial η^2^ = 0.074), group effect (*F*(1, 8) = 0.588, *p* = 0.4653, partial η^2^ = 0.068), or group-by-phase interaction (*F*(1.389, 11.11) = 1.778, *p* = 0.2136, partial η^2^ = 0.182; Figure 4c). Values were quite similar across phases in Control hearts (8.13 ± 0.82, 7.79 ± 0.71, and 7.80 ± 0.61 ms) and ISO-treated hearts (6.89 ± 0.83, 7.31 ± 0.94, and 6.87 ± 0.99 ms). Regional APD90 nevertheless showed a phase effect (*F*(1.581, 12.65) = 9.805, *p* = 0.0040, partial η^2^ = 0.551) and phase-by-region interaction (*F*(1.126, 9.010) = 5.621, *p* = 0.0390, partial η^2^ = 0.413), without a three-way interaction (*p* = 0.3884, partial η^2^ = 0.098; Table A1). Thus, modest regional APD90 changes did not translate into increased mapped-surface APD90 dispersion.

Among additional global timing metrics, total ventricular activation time and total CaT activation time showed no phase effect (*F*(1.197, 9.572) = 2.549, *p* = 0.1408, partial η^2^ = 0.242; and *F*(1.099, 8.795) = 1.688, *p* = 0.2296, partial η^2^ = 0.174, respectively). Global AP and CaT upstroke times showed phase effects (*F*(1.235, 9.877) = 7.907, *p* = 0.0151, partial η^2^ = 0.497; and *F*(1.258, 10.06) = 7.211, *p* = 0.0185, partial η^2^ = 0.474), without group-by-phase interactions. In the regional AP-upstroke model, group (*p* = 0.0060, partial η^2^ = 0.631), region (*p* = 0.0067, partial η^2^ = 0.622), and phase-by-region (*p* = 0.0124, partial η^2^ = 0.461) were significant, whereas the three-way interaction was not (*p* = 0.7140, partial η^2^ = 0.034). No effect reached *p* < 0.05 in the regional CaT-upstroke model (Table A1).

## 4. Discussion

Using simultaneous voltage and calcium optical mapping, this exploratory study of five hearts per group characterized response patterns across a fixed acute CCM sequence in Control hearts and ISO-treated hearts with features compatible with early remodeling. Optical AP and CaT ΔF/F amplitudes were approximately 5–6% above Base during CCM-ON and remained elevated during CCM-OFF in both groups. APD90 and CTD90 showed modest phase-dependent changes, and global CTD90 was longer overall in ISO-treated hearts. Formal regional models identified phase-by-region interactions for CTD90 and AP–CaT delay and a group-by-region interaction for CTD90, without significant three-way interactions. CTD90 spatial heterogeneity and the absolute proximal–distal difference in AP–CaT delay also varied by phase, whereas mapped-surface APD90 dispersion did not. Given the exploratory design and limited sample, these observations are intended to characterize response patterns and generate hypotheses rather than establish definitive group equivalence or clinical safety.

### 4.1. Functional Implications of Global Voltage and Calcium-Transient Responses During Acute CCM

Abnormal intracellular calcium regulation is a central feature of established HF and may also emerge during earlier stages of myocardial remodeling. Reduced sarcoplasmic reticulum (SR) calcium uptake, including alterations in the SERCA2a–phospholamban regulatory system, can contribute to diminished CaT amplitude, delayed calcium clearance, and impaired myocardial relaxation [3,7,18]. Previous experimental and translational studies have associated CCM with changes in calcium-regulatory protein expression, calcium-transient behavior, and myocardial contractile function [10,19,20,21].

From a translational perspective, SERCA2a is relevant because it resequesters cytosolic Ca^2+^ into the sarcoplasmic reticulum, thereby supporting diastolic relaxation and replenishing sarcoplasmic-reticulum Ca^2+^ stores for the subsequent contraction [3,18]. Chronic CCM improved sarcoplasmic-reticulum calcium-cycling proteins in a canine heart-failure model [19], whereas repeated CCM delivery over 7 days upregulated SERCA2a in a rabbit heart-failure model [22]. However, in the neutral CUPID 2 trial, AAV1/SERCA2a gene transfer at the dose tested did not improve the clinical course of patients with heart failure, illustrating that the biological plausibility of this pathway does not by itself guarantee clinical benefit [23]. Thus, the higher relative CaT amplitude and modest CTD90 shortening observed here are compatible with altered calcium cycling but do not demonstrate SERCA2a involvement, because SERCA2a expression or activity and phospholamban phosphorylation were not measured.

Calcium cycling is also coupled to myocardial energetics and redox homeostasis. ATP generated by mitochondrial oxidative phosphorylation supports SERCA-mediated Ca^2+^ reuptake into the sarcoplasmic reticulum [24]. The mitochondrial electron-transport chain is also a potential source of reactive oxygen species, and excessive mitochondrial ROS has been linked to ventricular arrhythmias and chronic proteome remodeling in heart failure [25]. Chronic CCM has been associated with improved myocardial metabolic remodeling in a rabbit heart-failure model [26] and with transcriptional changes in pathways related to mitochondrial structure, function, and cellular respiration in paired myocardial-biopsy RNA-sequencing data from six patients within a clinical study [27]. These findings provide a plausible link between CCM, cellular energetics, and structural or functional recovery; however, the present acute study did not measure mitochondrial respiration, ATP production, electron-transport-chain activity, or redox biomarkers, and no redox mechanism can therefore be inferred.

Optical CaT ΔF/F amplitude was approximately 6% above Base during CCM-ON and CCM-OFF, accompanied by modest phase-dependent shortening of CTD90. This pattern is broadly consistent with previous observations that acute CCM can alter CaT magnitude and recovery kinetics [20,21]. Representative waveforms were characterized predominantly by higher CaT signal amplitude and earlier recovery, whereas the voltage waveforms showed higher optical signal amplitude and broadening of the AP plateau. These observations are compatible with a larger optical CaT signal and faster calcium-transient recovery, but they do not establish increased SR calcium uptake or a change in absolute intracellular calcium concentration. Moreover, because no time-matched pacing-only sham session was included, the modest amplitude differences should be interpreted as phase-associated observations within the fixed sequence and cannot be attributed exclusively to CCM.

The voltage pattern differed in several respects from the calcium-transient pattern. Optical AP ΔF/F signal amplitude was approximately 5–6% above Base during CCM-ON and CCM-OFF, whereas APD90 showed only modest phase-dependent prolongation. These fluorescence differences should be interpreted as changes in optical signal amplitude rather than as direct measurements of transmembrane voltage or absolute intracellular calcium concentration. The modest APD90 prolongation observed in the present study also differs from previous reports of acute CCM-associated APD shortening [28]. This discrepancy may reflect differences in species, myocardial substrate, pacing rate, CCM waveform and timing, or electrophysiological recording methodology.

The broader AP plateau observed in representative waveforms may reflect secondary changes in membrane ionic currents coupled to altered intracellular calcium cycling. One possible contributor is altered electrogenic sodium–calcium exchange, through which changes in intracellular calcium dynamics may influence net membrane current during the plateau and repolarization phases [29]. This explanation remains a mechanistic hypothesis.

Several phase-associated differences remained evident during the brief CCM-OFF period. Optical AP and CaT signal amplitudes remained above their respective Base-phase values, and CTD90 remained significantly shorter than Base in Control hearts. Previous acute in vitro studies reported that CCM-related responses subsided after stimulation was stopped [20,30]. In contrast, the present CCM-OFF observations persisted over the brief post-stimulation interval. However, because no time-matched sham session was included, they cannot be distinguished from an experimental time trend or a combination of both. The mechanism and duration of the CCM-OFF pattern therefore remain unresolved.

Finally, the global CTD90 model showed a group main effect, indicating a longer average CTD90 in ISO-treated hearts across the protocol, without evidence of a different phase response because the group-by-phase interaction was not significant. The prespecified baseline Welch comparison was significant, whereas the three phase-specific comparisons did not individually reach significance after joint Šídák adjustment. Between-group mean differences in optical AP and CaT amplitude ratios were close to zero, but the 95% CIs for Hedges’ *g* were wide; these nonsignificant comparisons therefore do not establish equivalent amplitude responses between groups. No group-by-phase interaction was detected for APD90. The data support a persistent model-level CTD90 offset, but the small sample and wide phase-specific CIs preclude precise claims about each phase separately.

### 4.2. Proximity-Associated Regional Modulation of CTD90 and AP-CaT Timing

A central finding was that CTD90 exhibited both phase-by-region and group-by-region interactions. The phase-related decrease was greater proximally than distally, particularly in Control hearts, and the ISO-minus-Control difference was concentrated proximally during CCM-ON and CCM-OFF. The absence of a phase-by-group-by-region interaction indicates that the spatial phase pattern was not shown to differ between groups. These observations support a proximity-associated regional organization of CaT recovery while avoiding the invalid inference that significance in one region and nonsignificance in another alone proves a regional difference.

The observed proximity-associated pattern is biologically plausible given localized CCM delivery. Computational modeling has demonstrated spatial variation in simulated pacing fields surrounding myocardial CCM electrodes [12]. In isolated rabbit hearts, acute electrophysiological responses were localized near the stimulation site and varied with stimulation location and signal parameters [28]. Studies in human engineered cardiac tissues have likewise demonstrated parameter-dependent changes in contractile force and contraction–relaxation kinetics [30]. These observations collectively support the biological plausibility of a graded local myocardial response. Because local field strength was not measured or reconstructed here, the findings are described as proximity-associated rather than as evidence of a quantified field-strength-dependent effect.

AP–CaT delay also showed a phase-by-region interaction. Directed proximal-minus-distal estimates shifted in the negative direction during CCM-ON and CCM-OFF, consistent with a greater reduction proximally, although the CIs for individual phase-change contrasts were broad. Region-specific follow-up comparisons reached significance for proximal OFF-versus-Base in both groups, whereas ON-versus-Base did not. This nonuniform post hoc significance should not be interpreted as evidence that the response emerged only after stimulation ceased. Rather, the phase-by-region interaction and the direction of the estimates support a modest spatially nonuniform phase response whose precise timing requires confirmation.

AP–CaT delay aggregated across the mapped ventricular surface changed relatively little despite the phase-by-region interaction. This distinction illustrates how mapped-surface averaging can attenuate regional responses when changes differ by location. Regional optical mapping therefore provides information that may not be apparent from surface-aggregated measurements alone.

### 4.3. Spatial Dissociation Between Calcium-Related Heterogeneity and APD90 Dispersion During Acute CCM

Because altered intracellular calcium cycling can contribute to arrhythmogenic mechanisms [16], an important objective was to determine whether spatially nonuniform CaT recovery and AP–CaT timing responses were accompanied by increased ventricular repolarization dispersion. CTD90 spatial SD and the absolute proximal–distal difference in AP–CaT delay both showed phase effects and were numerically highest during CCM-ON. By contrast, APD90 spatial SD showed no detectable phase effect. These findings indicate a dissociation between calcium-transient recovery and AP–CaT timing on the one hand and mapped-surface APD90 dispersion on the other under the present experimental conditions.

One possible interpretation of this dissociation relates to differences in the spatial integration of intracellular calcium signaling and membrane voltage. Intracellular calcium cycling is regulated largely at cellular and subcellular levels and may therefore retain regional variation in response to localized stimulation. By contrast, electrotonic interactions between electrically coupled cardiomyocytes can modulate local repolarization differences [31]. However, recent intact-heart optical mapping indicates that APD heterogeneity is also shaped by endogenous APD gradients, activation sequence, stimulus effects, and the method used to quantify dispersion [32]. The absence of a detectable increase in APD90 spatial SD may therefore reflect the combined influence of myocardial electrical integration and the underlying spatial distribution of APD90, rather than electrotonic coupling alone. These mechanisms were not directly tested and remain hypotheses.

From an arrhythmogenic perspective, the relevant observation is not that acute CCM was devoid of potential proarrhythmic effects, but that increased calcium-related spatial heterogeneity was not accompanied by a parallel detectable increase in mapped-surface APD90 dispersion. APD90 dispersion represents only one component of arrhythmia susceptibility. Local conduction velocity, conduction block, restitution, electrical and calcium alternans, triggered activity, and arrhythmia inducibility were not assessed. The present finding should therefore be interpreted as evidence concerning repolarization heterogeneity rather than as establishing overall electrophysiological safety.

#### Limitations

This study has several limitations. First, the experiments were performed using an ex vivo Langendorff-perfused heart preparation to enable high-resolution simultaneous optical mapping of membrane-voltage and calcium-transient signals under tightly controlled conditions. Although this platform reduces systemic confounding, it does not preserve autonomic regulation, circulating neurohumoral influences, physiological ventricular loading, or normal in vivo metabolic conditions. Blebbistatin was used to suppress motion artifacts, precluding direct assessment of myocardial contraction, ventricular mechanics, and global hemodynamic performance. Optical measurements were also restricted to the mapped ventricular surface and therefore did not capture intramural or endocardial electrophysiological gradients. In addition, programmed arrhythmia provocation and direct assessment of triggered activity, electrical alternans, and restitution properties were not performed. The present findings should therefore be interpreted as acute mapped-surface myocardial responses in an isolated-heart preparation rather than as integrated in vivo physiological or arrhythmia outcomes.

Second, the optical amplitude measurements require cautious interpretation. RH237- and Rhod-2-derived amplitudes represent relative fluorescence changes rather than direct measurements of transmembrane voltage or absolute intracellular calcium concentration. Optical AP and CaT amplitudes during CCM-ON and CCM-OFF were further expressed relative to the corresponding Base-phase value within each heart. A separate time-matched pacing-only sham-stimulation session was not included. Although within-heart normalization reduces between-heart variability, it does not control for time-dependent experimental confounding, including temporal drift, dye bleaching or redistribution, phototoxicity, or gradual changes in signal stability. The persistence of higher amplitudes during CCM-OFF could therefore reflect a short-lived biological after-effect, an experimental time trend, or both. Accordingly, the modest phase-associated amplitude differences of approximately 5–6% observed during CCM-ON and CCM-OFF cannot be attributed exclusively to CCM.

Third, the study was designed to characterize acute spatiotemporal responses rather than to define the underlying molecular mechanisms or long-term biological effects of CCM. SERCA2a activity, phospholamban phosphorylation, function of sodium–calcium exchanger, gap-junction conductance, and individual membrane ionic currents were not directly examined. Local electric-field strength was also neither measured nor computationally reconstructed; the regional findings should therefore be interpreted as proximity-associated responses rather than as direct evidence of a quantified field-strength effect. Furthermore, persistence was assessed only during a brief post-stimulation interval, and whether the observed responses are maintained during chronic CCM or contribute to reverse remodeling remains unknown.

Fourth, this exploratory mechanistic study had a limited sample size. Repeated-measures analyses and within-heart comparisons improved efficiency, but modest effects and higher-order interactions may have remained undetected. Region was incorporated as a repeated factor in the three-way models, directly testing phase-by-region and group-by-region effects; nevertheless, the three-way interaction estimates and several directed regional contrasts had wide CIs. Nonsignificant interactions or between-group comparisons should therefore not be interpreted as evidence of equivalent CCM responsiveness. Multiple global, regional, and dispersion-related metrics were examined. Although prespecified comparisons were corrected within their respective families, no single correction was applied across the full set of metrics analyzed in the study. The findings should accordingly be regarded as exploratory and hypothesis-generating and require confirmation in larger independent cohorts.

Finally, ISO-treated hearts exhibited qualitative structural alterations, prolonged CTD90, and numerically longer activation-related metrics, representing features compatible with early remodeling. However, comprehensive in vivo phenotyping, including echocardiography, ventricular mass indices, quantitative histomorphometry, and longitudinal functional assessment, was not performed. The model should therefore be regarded as an ISO-treated phenotype with features compatible with early remodeling rather than as a comprehensive or stage-specific model of early HF.

## 5. Conclusions

In conclusion, this exploratory intact-heart study identified spatially nonuniform phase-associated patterns in CaT recovery and AP–CaT timing across a fixed acute CCM sequence in Control hearts and ISO-treated hearts with features compatible with early remodeling, without a detectable increase in APD90 dispersion across the mapped ventricular surface. The findings support a spatial dissociation between calcium-related temporal responses and mapped-surface APD90 dispersion under the present experimental conditions. Because APD90 dispersion captures only one aspect of arrhythmia susceptibility and arrhythmia susceptibility was not directly assessed, this dissociation should not be interpreted as evidence that acute CCM lacks proarrhythmic effects or is electrophysiologically safe. Given the small sample and absence of time-matched sham stimulation, these findings should be considered hypothesis-generating, and causal interpretation is particularly limited for the modest normalized optical amplitude differences. Future work should first determine whether these response patterns are reproducible and distinguishable from protocol-related time trends in larger cohorts incorporating a time-matched sham condition. Once the acute responses are confirmed, future studies could characterize local electric-field strength and assess its relationship with electrical propagation, mechanical function, and arrhythmia susceptibility. Additional experiments could examine whether the regional optical patterns are linked to SERCA2a-related calcium handling, myocardial energetics, redox homeostasis, and longer-term reverse remodeling.

## Data Availability

The original contributions presented in this study are included in the article. Further inquiries can be directed to the corresponding authors.

## Abbreviations

The following abbreviations are used in this manuscript:

AP	Action potential
APD90	Action potential duration at 90% repolarization
CCM	Cardiac contractility modulation
CaT	Calcium transient
CTD90	Calcium transient duration at 90% recovery
HF	Heart failure
ISO	Isoproterenol
SERCA2a	Sarcoplasmic reticulum Ca^2+^ATPase 2a
SR	Sarcoplasmic reticulum

## Appendix A

**Table A1:** Additional global and regional optical-mapping metrics across the Base, CCM-ON, and CCM-OFF phases. Data are mean ± SEM (*n* = 5 hearts per group). Panel A metrics were analyzed using two-way repeated-measures ANOVA with group as the between-subject factor and phase as the within-subject factor. Panel B metrics were analyzed using three-way repeated-measures ANOVA with group as the between-subject factor and phase and region as within-subject factors. In $F(df_{num},\;{df}_{den})$, $df_{num}$ and ${df}_{den}$ denote the numerator and denominator degrees of freedom, respectively. Greenhouse–Geisser-corrected degrees of freedom and exact *p* values are reported for effects involving phase when applicable. Partial η2 was calculated for every ANOVA effect. AP, action potential; APD90, action-potential duration at 90% repolarization; CaT, calcium transient; CCM, cardiac contractility modulation; SEM, standard error of the mean.

**Panel A. Additional global mapping metrics**					
**Descriptive statistics**					
**Measure**	**Group**		**Base**	**CCM-ON**	**CCM-OFF**
Total ventricular activation time (ms)	Control		22.89 ± 0.64	22.17 ± 0.89	23.01 ± 0.57
	ISO		25.72 ± 1.24	25.13 ± 1.36	25.93 ± 1.40
Global AP upstroke time (ms)	Control		8.79 ± 0.10	8.83 ± 0.14	8.97 ± 0.11
	ISO		9.38 ± 0.25	9.31 ± 0.20	9.56 ± 0.25
Total CaT activation time (ms)	Control		24.51 ± 1.00	23.82 ± 1.33	24.54 ± 0.87
	ISO		27.32 ± 1.69	26.65 ± 1.55	27.00 ± 1.70
Global CaT upstroke time (ms)	Control		10.67 ± 0.17	10.65 ± 0.15	10.57 ± 0.14
	ISO		10.87 ± 0.32	10.72 ± 0.25	10.70 ± 0.27
Global AP–CaT delay (ms)	Control		6.00 ± 0.25	5.58 ± 0.50	5.92 ± 0.24
	ISO		6.50 ± 0.49	6.42 ± 0.65	6.75 ± 0.61
**Two-way repeated-measures ANOVA results**					
**Measure**	**Effect**		***F* (df_num_, df_den_)**	**Exact *p***	**Partial η^2^**
Total ventricular activation time (ms)	Group		*F*(1, 8) = 4.045	0.0791	0.336
	Phase		*F*(1.197, 9.572) = 2.549	0.1408	0.242
	Group × Phase		*F*(1.197, 9.572) = 0.01377	0.9384	0.002
Global AP upstroke time (ms)	Group		*F*(1, 8) = 4.576	0.0649	0.364
	Phase		*F*(1.235, 9.877) = 7.907	0.0151	0.497
	Group × Phase		*F*(1.235, 9.877) = 0.6255	0.4805	0.073
Total CaT activation time (ms)	Group		*F*(1, 8) = 1.976	0.1974	0.198
	Phase		*F*(1.099, 8.795) = 1.688	0.2296	0.174
	Group × Phase		*F*(1.099, 8.795) = 0.1422	0.7387	0.017
Global CaT upstroke time (ms)	Group		*F*(1, 8) = 0.1731	0.6883	0.021
	Phase		*F*(1.258, 10.06) = 7.211	0.0185	0.474
	Group × Phase		*F*(1.258, 10.06) = 1.59	0.2428	0.166
Global AP–CaT delay (ms)	Group		*F*(1, 8) = 1.25	0.2961	0.135
	Phase		*F*(1.389, 11.12) = 1.529	0.2521	0.160
	Group × Phase		*F*(1.389, 11.12) = 0.4706	0.5687	0.056
**Panel B. Additional regional mapping metrics**					
**Descriptive statistics**					
**Measure**	**Region**	**Group**	**Base**	**CCM-ON**	**CCM-OFF**
AP upstroke time (ms)	Proximal	Control	8.49 ± 0.09	8.25 ± 0.16	8.63 ± 0.11
		ISO	9.39 ± 0.23	9.08 ± 0.20	9.60 ± 0.26
	Distal	Control	8.72 ± 0.20	8.87 ± 0.16	8.86 ± 0.13
		ISO	9.73 ± 0.25	9.81 ± 0.30	9.77 ± 0.30
CaT upstroke time (ms)	Proximal	Control	10.39 ± 0.13	10.33 ± 0.14	10.34 ± 0.11
		ISO	10.90 ± 0.21	10.92 ± 0.16	10.82 ± 0.15
	Distal	Control	10.67 ± 0.13	10.73 ± 0.15	10.52 ± 0.06
		ISO	10.78 ± 0.27	10.74 ± 0.20	10.68 ± 0.24
APD90 (ms)	Proximal	Control	82.43 ± 6.79	85.17 ± 6.78	85.09 ± 6.80
		ISO	83.15 ± 5.65	88.57 ± 5.61	86.28 ± 4.46
	Distal	Control	80.65 ± 4.66	86.04 ± 4.86	83.87 ± 5.21
		ISO	82.59 ± 4.91	89.29 ± 4.25	86.31 ± 3.34
**Three-way repeated-measures ANOVA results**					
**Measure**	**Effect**		***F* (df_num_, df_den_)**	**Exact *p***	**Partial η^2^**
AP upstroke time (ms)	Group		*F*(1, 8) = 13.7	0.0060	0.631
	Phase		*F*(1.461, 11.69) = 3.786	0.0644	0.321
	Region		*F*(1, 8) = 13.18	0.0067	0.622
	Group × Phase		*F*(1.461, 11.69) = 0.102	0.8443	0.013
	Group × Region		*F*(1, 8) = 0.06462	0.8057	0.008
	Phase × Region		*F*(1.62, 12.96) = 6.83	0.0124	0.461
	Group × Phase × Region		*F*(1.62, 12.96) = 0.2815	0.7140	0.034
CaT upstroke time (ms)	Group		*F*(1, 8) = 2.269	0.1704	0.221
	Phase		*F*(1.696, 13.57) = 2.32	0.1409	0.225
	Region		*F*(1, 8) = 0.5989	0.4612	0.070
	Group × Phase		*F*(1.696, 13.57) = 0.01423	0.9754	0.002
	Group × Region		*F*(1, 8) = 5.104	0.0538	0.389
	Phase × Region		*F*(1.202, 9.613) = 0.462	0.5477	0.055
	Group × Phase × Region		*F*(1.202, 9.613) = 0.8796	0.3918	0.099
APD90 (ms)	Group		*F*(1, 8) = 0.08654	0.7761	0.011
	Phase		*F*(1.581, 12.65) = 9.805	0.0040	0.551
	Region		*F*(1, 8) = 0.04829	0.8316	0.006
	Group × Phase		*F*(1.581, 12.65) = 0.406	0.6281	0.048
	Group × Region		*F*(1, 8) = 0.06951	0.7987	0.009
	Phase × Region		*F*(1.126, 9.01) = 5.621	0.0390	0.413
	Group × Phase × Region		*F*(1.126, 9.01) = 0.8716	0.3884	0.098
